# Correction: MiR-940 inhibits TGF-β-induced epithelial-mesenchymal transition and cell invasion by targeting Snail in non-small cell lung cancer

**DOI:** 10.7150/jca.48686

**Published:** 2020-06-11

**Authors:** Kanqiu Jiang, Ting Zhao, Mingjing Shen, Fuquan Zhang, Shanzhou Duan, Zhe Lei, Yongbing Chen

**Affiliations:** 1 Department of Thoracic and Cardiovascular Surgery, The Second Affiliated Hospital of Soochow University, Medical College of Soochow University, Suzhou 215004, China; 2 Soochow University Laboratory of Cancer Molecular Genetics, Medical College of Soochow University, Suzhou 215123, China; 3 Department of Genetics, School of Biology and Basic Medical Science, Medical College of Soochow University, Suzhou, Jiangsu, 215123, China

In the initially published version of this article, the representative images of the invasion test of A549 cells overexpressing miR-940 with or without TGF-β1 stimulation in Figure 3C are wrong. The correct Figure 3C is as follows:

The correction made in this erratum does not affect the counting results and original conclusions. The authors apologize for any inconvenience or misunderstanding that this error may have caused.

## Figures and Tables

**Figure 3:**
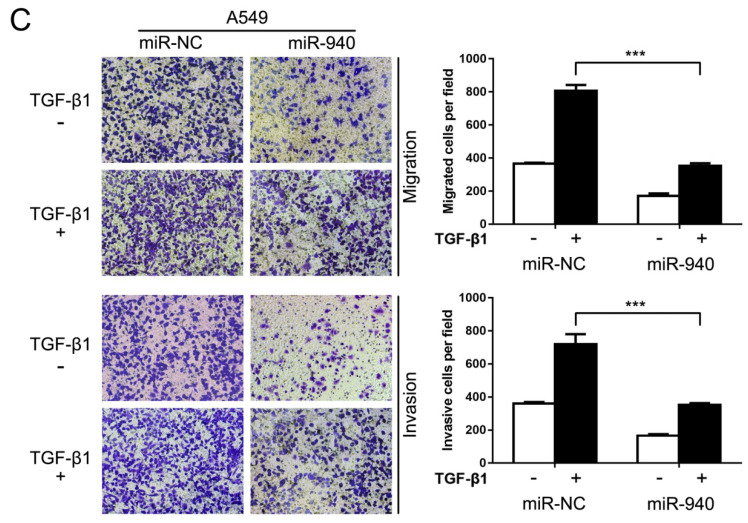
(C) Transwell assays for A549 cells transfected with miR-NC or miR-940 mimics in the absence or presence of TGF-β1. Migrated and invaded cells were stained and counted in at least three microscopic fields (magnification ×100).

## References

[1] Jiang K, Zhao T, Shen M, Zhang F, Duan S, Lei Z, Chen Y (2019). MiR-940 inhibits TGF-β-induced epithelial-mesenchymal transition and cell invasion by targeting Snail in non-small cell lung cancer. J Cancer.

